# Reduced paediatric hospitalizations for malaria and febrile illness patterns following implementation of community-based malaria control programme in rural Rwanda

**DOI:** 10.1186/1475-2875-7-167

**Published:** 2008-08-27

**Authors:** Amy C Sievers, Jenifer Lewey, Placide Musafiri, Molly F Franke, Blaise J Bucyibaruta, Sara N Stulac, Michael L Rich, Corine Karema, Johanna P Daily

**Affiliations:** 1 Brigham and Women's Hospital, Harvard Medical School, Boston, MA, USA; 2 Rwinkwavu Hospital, Partners in Health, Rwinkwavu, Rwanda; 3 Harvard Medical School, Boston, MA, USA; 4 Programme National Intégré de Lutte contre le Paludisme, Kigali, Rwanda

## Abstract

**Background:**

Malaria control is currently receiving significant international commitment. As part of this commitment, Rwanda has undertaken a two-pronged approach to combating malaria via mass distribution of long-lasting insecticidal-treated nets and distribution of antimalarial medications by community health workers. This study attempted to measure the impact of these interventions on paediatric hospitalizations for malaria and on laboratory markers of disease severity.

**Methods:**

A retrospective analysis of hospital records pre- and post-community-based malaria control interventions at a district hospital in rural Rwanda was performed. The interventions took place in August 2006 in the region served by the hospital and consisted of mass insecticide treated net distribution and community health workers antimalarial medication disbursement. The study periods consisted of the December–February high transmission seasons pre- and post-rollout. The record review examined a total of 551 paediatric admissions to identify 1) laboratory-confirmed malaria, defined by thick smear examination, 2) suspected malaria, defined as fever and symptoms consistent with malaria in the absence of an alternate cause, and 3) all-cause admissions. To define the impact of the intervention on clinical markers of malaria disease, trends in admission peripheral parasitaemia and haemoglobin were analyzed. To define accuracy of clinical diagnoses, trends in proportions of malaria admissions which were microscopy-confirmed before and after the intervention were examined. Finally, to assess overall management of febrile illnesses antibiotic use was described.

**Results:**

Of the 551 total admissions, 268 (48.6%) and 437 (79.3%) were attributable to laboratory-confirmed and suspected malaria, respectively. The absolute number of admissions due to suspected malaria was smaller during the post-intervention period (N = 150) relative to the pre-intervention period (N = 287), in spite of an increase in the absolute number of hospitalizations due to other causes during the post-intervention period. The percentage of suspected malaria admissions that were laboratory-confirmed was greater during the pre-intervention period (80.4%) relative to the post-intervention period (48.1%, prevalence ratio [PR]: 1.67; 95% CI: 1.39 – 2.02; chi-squared p-value < 0.0001). Among children admitted with laboratory-confirmed malaria, the risk of high parasitaemia was higher during the pre-intervention period relative to the post-intervention period (age-adjusted PR: 1.62; 95% CI: 1.11 – 2.38; chi-squared p-value = 0.004), and the risk of severe anaemia was more than twofold greater during the pre-intervention period (age-adjusted PR: 2.47; 95% CI: 0.84 – 7.24; chi-squared p-value = 0.08). Antibiotic use was common, with 70.7% of all children with clinical malaria and 86.4% of children with slide-negative malaria receiving antibacterial therapy.

**Conclusion:**

This study suggests that both admissions for malaria and laboratory markers of clinical disease among children may be rapidly reduced following community-based malaria control efforts. Additionally, this study highlights the problem of over-diagnosis and over-treatment of malaria in malaria-endemic regions, especially as malaria prevalence falls. More accurate diagnosis and management of febrile illnesses is critically needed both now and as fever aetiologies change with further reductions in malaria.

## Background

Combating malaria is currently the target of an impressive resurgence in international commitment, in particular in sub-Saharan Africa where the burden of disease is greatest [1,2]. International institutions such as the World Health Organization and the Global Fund to Fight AIDS, Tuberculosis and Malaria, national commitments such as The President's Malaria Initiative, and private organizations such as the Bill and Melinda Gates Foundation, have raised awareness and dedicated substantial financial and technical support to malaria control efforts. There have been encouraging early reports on the results of these efforts, which in turn have helped further the case for more investment in malaria control [3]. Indeed, the Gates Foundation's recent call for malaria eradication might have seemed unthinkable only a few years ago [4].

Investments are being made in all areas of malaria control: vaccine and drug development, vector control, infrastructure development, and improving service delivery and accessibility where it is most needed. Two approaches with potential for immediate results are 1) exposure reduction through long-lasting insecticide-treated bed nets (LLINs), indoor residual spraying, and larvicides, and 2) early treatment using community health workers. Multiple studies have demonstrated the efficacy of these community-level interventions in terms not only of reducing episodes of malaria but improving child survival as a whole [5,6]. For example, increasing LLIN coverage in Kenya from 6% to 67% correlated with a 44% reduction in overall child mortality [7]. Early treatment through Home-Based Management (HBM) of malaria is a key strategy supported by Roll Back Malaria (RBM) [8]. RBM's HBM programme is designed to decrease barriers to children receiving appropriate antimalarial therapy in a timely manner and relies on a workforce without formal medical training, such as community health workers and primary caregivers, to identify illness and provide care. Given the relatively recent implementation of the HBM strategy, limited outcomes data exist at present. However, early reports are encouraging in terms of both results and adherence [9-11]. Heavy use of chloroquine has affected early outcome reporting, and the broad transition across sub-Saharan Africa to artemisinin combination therapies (ACTs) for HBM programmes is anticipated to result in even greater successes [12].

Rwanda has undertaken an aggressive community-based prevention and early treatment strategy as part of its national malaria control programme with excellent preliminary results, including a 66% reduction in childhood deaths attributed to malaria [5]. The primary interventions are mass distribution of LLINs to pregnant women and children under five years of age and distribution of antimalarial medications by community health workers. Additionally, Rwanda adopted ACTs as first line treatment for uncomplicated malaria at health centres and hospitals as a slow rollout beginning in early 2006. This study attempts to measure the impact of LLIN and community-based medication distribution on outcomes other than mortality by examining changes in hospitalization patterns for malaria and changes in markers of disease severity at a rural district hospital, before and after implementation of the control programme.

## Methods

### Study design and rationale

To determine the effect of a community-based malaria prevention and early treatment programme, a retrospective analysis of paediatric hospital admissions records before and after the intervention was carried out. Study aims were to determine whether there was a reduction in the proportion of laboratory-confirmed clinical malaria admissions, among children who were admitted for malaria, whether there was an improvement in clinical markers of malaria disease, specifically haemoglobin and peripheral parasitaemia. This study was approved by the Rwandan National Ethical Committee as well as by the Institutional Review Board at Brigham and Women's Hospital, Harvard Medical School.

### Study centre and patients

This study was conducted at Rwinkwavu Hospital, the district hospital for Southern Kayonza, a rural region in the Eastern Province of Rwanda. The hospital is jointly managed by the Ministry of Health, Partners in Health (PIH), and the Clinton Foundation. Rwinkwavu Hospital serves as the referral hospital for regions covered by seven affiliated health centres in Southern Kayonza. In addition, patients requiring blood transfusions were also referred to Rwinkwavu Hospital from a neighbouring district during the study periods. Southern Kayonza is located in a malaria-endemic zone with two high transmission seasons, December through February and April through June, and with sporadic cases arising throughout the year.

The study was carried out over two consecutive malaria high transmission seasons pre-intervention (December 2005 through February 2006) and post-intervention (December 2006 through February 2007). The eligible study population included all children admitted to the paediatric ward. Data for admitted children are entered into a discharge registry, which is maintained by nursing staff and includes basic demographic information, admission diagnosis, clinical course and management, discharge diagnosis, and clinical outcome. Children for whom there was no discharge diagnosis in the discharge registry were excluded from this study.

### Description of intervention

The Rwandan Ministry of Health, supported by PIH and the Clinton Foundation, embarked on an intensive community-based prevention and early treatment malaria control programme. The prevention component was based on mass distribution of LLINs. Beginning in March 2006, nets were distributed by Rwinkwavu Hospital and its affiliated health centres to pregnant women as part of routine antenatal care, to all hospitalized children, to all malnourished children, and to many patients with HIV. Additionally, the Ministry of Health, supported by the Global Fund to Fight AIDS, Tuberculosis and Malaria, organized mass distribution of LLINs to children of five years of age or less in September 2006 as part of an integrated measles vaccine campaign. A total of over 26,000 nets were distributed in southern Kayonza, an area with approximately 28,000 individual dwellings and a total population of 130,000. The majority of LLINs were distributed via the vaccination campaign. The early treatment component consisted of 300 community health workers (CHWs), who were trained to distribute antimalarials within each village to children of five years of age or less with fever and symptoms consistent with uncomplicated malaria. Additionally, CHWs were trained to identify and refer children with more severe disease, and poor po intake to their local health centres. Children who were considered more severely ill or required IV hydration by health centre clinical staff based on their clinical judgement were in turn transferred to Rwinkwavu Hospital. Finally, in December of 2006, we conducted a series of staff training programmes aimed at improving hospital-based paediatrics care and malaria care in particular. This included more rigorous guidelines for laboratory monitoring, including checking admission haemoglobin for all children with suspected malaria.

Between rollout in September 2006 and study end in February 2007, 11,390 children were treated by CHWs within the communities served by the seven health centres affiliated with Rwinkwavu Hospital, and 1,408 (12%) were referred to a higher level of care. Initial training and drug supply was provided by the national malaria control programme (Programme National Intégré de Lutte contre le Paludisme, PNILP). Subsequent training, support and monitoring were provided by PIH staff and data were reported to PNILP. All therapeutic regimens were in accordance with Rwanda Ministry of Health guidelines. Drug supply was supported by the Global Fund to Combat AIDS, Tuberculosis and Malaria. The community-based treatment regimen consisted of age-based blister packs of sulphadoxine-pyrimethamine + amodiaquine (SP+AQ). Hospitalized children received intravenous quinine until able to tolerate oral medications, at which point they were switched to oral quinine to complete a 7 day course of treatment. Health centres were using AL for uncomplicated malaria but this predated the onset of the study period.

### General study procedures

Discharge registries were retrospectively reviewed to classify each child as either a malaria or non-malaria admission and obtain basic clinical and demographic data. Suspected malaria admission was defined as being given a discharge diagnosis of malaria in the registry. Suspected diagnosis of malaria was made in children presenting with fever and symptoms consistent with malaria in the absence of an alternative diagnosis. Symptoms and findings suggestive of an alternative diagnosis included productive cough, chest radiograph findings consistent with pneumonia or tuberculosis, meningeal signs with positive lumbar puncture, or bloody diarrhea with abdominal pain. Even if children had a positive malaria smear, they were not given a primary diagnosis of malaria if their clinical diagnosis was an alternative febrile illness. Laboratory-confirmed malaria was defined as suspected malaria plus microscopic slide analysis demonstrating peripheral parasitaemia. To assess severity of malaria disease, the degree of admission peripheral parasitaemia and haemoglobin were examined. Peripheral parasitaemia was determined by examination of thick smears and scored on a 1+ to 4+ scale. On this scale, 1+ is defined as 1–10 trophozoites per 100 high power fields (hpf), 2+ as 11–100 trophozoites per 100 hpf, 3+ as 1–19 trophozoites per single hpf and 4+ as >11 trophozoites per single hpf. Parasitaemia was defined solely by the number of parasites per field and was not standardized to white count. Haemoglobin was measured from a venipuncture using QBC Autoread Plus (QBC Diagnostics, Philipsburg PA). Severe anemia was defined using WHO criteria ≤ 5 g/dl [13]. Data related to antibiotic and antimalarial use were also recorded. In cases where information was missing from the discharge registry, individual patient charts and laboratory records were examined. To examine whether changes in overall hospitalization patterns could account for trends in malaria hospitalizations, all-cause admission diagnoses during the pre- and post-intervention study periods were examined. Finally, rainfall and temperature data were collected from the Ministry of Infrastructure to assess potential environmental factors that may have altered transmission intensity and thus impacted the results.

### Statistical analysis

The primary outcome was the proportion of suspected malaria cases that were laboratory-confirmed, and this proportion was compared across the pre- and post-intervention periods. To examine whether there was an association between the intervention and markers of clinical disease, the proportions of children with laboratory-confirmed malaria who had a high peripheral parasitaemia (3+ or 4+) or severe anemia (haemoglobin < 5 g/dl) during the pre- and post-intervention periods were compared. Age-adjusted prevalence ratios, using Mantel-Haenszel weights, were calculated to account for potential confounding by age. Local climate data were obtained and qualitatively examined to determine whether differences in temperature or rainfall during the study periods could have confounded the relationship between the intervention and malaria outcomes. Data were analysed using SAS *version *9.12 (The SAS Institute, Cary, North Carolina).

## Results

A total of 554 paediatric admissions were recorded in the discharge registry during the two study periods. Three children did not have a discharge diagnosis and were excluded from the analysis. Of the remaining 551 admissions, 322 (58.4%) occurred during the pre-intervention period and 229 (41.6%) occurred during the post-intervention period. Table 1 and 2 report the absolute reductions in both suspected and laboratory-confirmed malaria between the pre- and post- intervention periods. Baseline characteristics of the enrolled children are shown in Table 1. The gender distribution of admitted children was comparable across study periods; however, age distributions differed, with children in the post-intervention tending to fall in older age categories (chi-squared p-value = 0.0006) (Table 1).

**Table 1:** Patient characteristics and admissions data

	**n (%)**			
		
**PATIENT CHARACTERISTICS**	**Pre**	**Post**	**Total**	**P value**
Gender (N = 539)				
F	161 (50.9)	110 (49.3)	271 (50.3)	0.71
Age (years) (N = 538)				
<1	143 (45.0)	79 (35.9)	222 (41.2)	
1 to 5	140 (44.0)	90 (40.9)	230 (42.8)	0.0006
>5	35 (11.0)	51 (23.2)	86 (16.0)	
**ADMISSION DIAGNOSES**				
Total Admissions	322	229	551	
**Total Suspected Malaria Admissions**	287 (89.1)	150 (65.5)	437 (79.3)	
**Total Other-Cause Admissions**	35 (10.9)	79 (34.5)	114 (20.6)	
Gastrointestinal infections	18 (51.4)	17 (21.5)	35 (30.7)	
Trauma/burns/bites	5 (14.3)	15 (19.0)	20 (17.5)	
Skin and soft tissue infections	2 (5.7)	11 (13.9)	13 (11.4)	
Respiratory infections	1 (2.9)	11 (13.9)	12 (10.5)	
Other infections	2 (5.7)	9 (11.4)	11 (9.6)	
CHF	0 (0.0)	6 (7.6)	6 (5.3)	
Neoplastic disease	1 (2.9)	2 (2.5)	3 (2.6)	
Meningitis	0 (0.0)	3 (3.9)	3 (2.6)	
TB	1 (2.9)	0 (0.0)	1 (0.9)	
HIV complications	1 (2.9)	0 (0.0)	1 (0.9)	
Other	4 (11.4)	5 (6.3)	9 (7.9)	

Pre- and post-intervention patient demographic and admissions data are listed. There were 13 children (4 pre- and 9 post-intervention) for whom age was not available. There were 12 children (6 pre- and 6 post-intervention) for whom gender was not available. Percents are recorded as proportion of the pre-intervention total, post-intervention total, or overall admissions total. CHF-congestive heart failure; TB-tuberculosis.

**Table 2:** Laboratory markers of malaria disease severity

	**n (%)**			
			
	**Pre**	**Post**	PR* [95% CI]	**chi-squared** ** p-value**
Slide-positive (N = 386)	205 (80.4)	63 (48.1)	1.67 [1.39–2.02]	<0.0001
Suspected malaria, no slide results available (N = 51)	32 (11.1)	19 (12.7)	0.88 [0.52–1.50]	0.64
High parasitaemia (N = 268)	116 (56.6)	22 (34.9)	1.62** [1.11–2.38]	0.004
Severe anemia, slide-positive admissions (N = 161)	21 (18.9)	4 (8.0)	2.47** [0.84–7.24]	0.08
Severe anemia, all hospitalizations (N = 307)	23 (15.3)	6 (3.8)	3.85** [1.60–9.25]	0.001
No hemoglobin result (N = 244)	172 (53.4)	72 (31.4)	1.70 [1.37–2.11]	<0.0001

Results for laboratory-definable markers of disease severity: admission haemoglobin and admission peripheral parasitaemia are shown. For parasitaemia, percents are recorded as proportion of pre-intervention, post-intervention, or total admissions for malaria for which a slide result was available. For low vs. high (3+-4+) parasitaemia, percents are recorded as proportion of total slide-positive malaria admissions. For missing data, percentages are recorded as proportion of total malaria admissions for which slide results were unavailable. Severe anemia is defined as ≤ 5 g/dl. * Prevalence ratio comparing the proportion in the pre-intervention period to the proportion in the post-intervention period. ** adjusted for age using three categories (<1 year; 1–5 years; > 5 years)

Fifty-one of 437 (11.7%) children admitted with suspected malaria lacked smear results (Table 1), and the percentage of children missing a smear result was similar for the pre- and post-intervention periods (11.1% and 12.6%, respectively; chi-squared p-value: 0.64). Among the 386 children with suspected malaria for whom smear results were available (Table 1), the percentage of suspected malaria admissions that were laboratory-confirmed was significantly higher in the pre-intervention period (80.4%) than during the post-intervention period (48.1%, prevalence ratio [PR]: 1.67; 95% CI: 1.39 – 2.02; chi-squared p-value < 0.0001). Relative to the pre-intervention period, children diagnosed with laboratory-confirmed malaria in the post-intervention period more frequently demonstrated laboratory markers of milder illness (Table 2). Table 2 displays admission peripheral parasitaemia between the two study periods. The percentage of children with a laboratory-confirmed diagnosis who had a high parasitaemia decreased significantly from the pre-intervention (56.6%) to the post-intervention period (34.9%; age-adjusted PR: 1.62; 95% CI: 1.11 – 2.38; chi-squared p-value = 0.004). The prevalence of severe anemia was also higher in the pre-intervention period among children with laboratory-confirmed malaria (age-adjusted PR: 2.47; 95% CI: 0.84 – 7.24; chi-squared p-value = 0.08) and among all children admitted to the paediatric ward (age-adjusted PR: 3.85; 95% CI: 1.60 – 9.25; chi-squared p-value = 0.001); however hospitalized children in the pre-intervention period were significantly more likely to lack a haemoglobin test result than children in the post-intervention period (53.4% vs. 31.4%, respectively; chi-squared p < 0.0001).

There were no deaths attributed to malaria in the pre-intervention period. There were two children whose deaths were attributed to malaria in post-intervention period, both of whom had negative thick smears. Across the years, 309 of the 437 children with suspected malaria (70.7%) were treated with antibiotics as well as antimalarials. Among 118 children with suspected malaria but negative thick smears, 102 (86.4%) were treated with antibiotics. Climate data obtained from the national weather station in Kayonza, including rainfall and temperature recordings, are reported in Table 3. Data for February 2007 were not available, so this report is restricted to the months of December and January pre- and post-intervention.

**Table 3:** Climate data

	**December 2005–January 2006**	**December 2006–January 2007**
	
Average daily high temperature (°C)	24.29	25.22
Average daily low temperature (°C)	7.04	3.99
Average daily temperature (°C)	15.67	14.6
Cumulative rainfall (cm)	135	127
Average daily humidity (%)	68.79	74.9

Temperature, humidity, and rainfall data for December and January of the pre-intervention and post-intervention periods are shown. Data were obtained from the national weather station in Kayonza, the weather station closest to Rwinkwavu. Results were not available for February 2007 so these data are limited to December and January in the pre- and post-intervention periods.

## Discussion

This analysis demonstrates a significant reduction in hospitalizations of children with malaria and in those children who were hospitalized with malaria, a decrease in parasite burden and anemia following rollout of community-based prevention and early treatment programmes. Although this study addresses only the short-term results of new malaria control measures, it is encouraging to observe such a measurable impact after a brief period of intervention.

Malaria intervention programs are undertaken in areas with unique disease patterns and utilization strategies, thus individual outcome studies are needed. Our goal therefore was to analyze routinely collected hospitalization records to assess malaria control interventions as this approach can be used widely and does not require additional resources. Retrospective, non population based analysis is an imperfect method to assess malaria control programmes in a community. There is no control group, those that present to the hospital may not completely represent disease prevalence at the community level and unforeseen factors can have impacted the outcomes, however hospitalization records can reflect major causes of mortality in a region [14]. The study hospital served the region that received bednet distribution and CHW treatment of malaria. Future CHW assessment of malaria in the villages, using RDTs with hospitalization data could provide stronger analytic capacity and result in cost effective treatment [15]. The study definitions of malaria disease severity were based solely on laboratory indicators. This provides objective data, however the prevalence of other important clinical manifestations of disease severity such as coma, respiratory distress or other accepted severe malaria syndromes were not captured [13]. Through training and improvement of the medical record template, these syndromes can be more routinely assessed. Parasitaemia was utilized as an indicator of more serious clinical disease. In areas of low transmission it can predict more severe outcomes, whereas in other regions it is not associated with more severe disease [13]. More careful assessment of all clinical manifestations of malaria and parasite burden would need to be studied to determine if this is a valid marker of severe disease in this region.

More comprehensive evaluation of long-term impact will require continued monitoring. One consequence of the control of malaria is that laboratory-confirmed disease comprised a much smaller percentage of malaria-like clinical disease in the post-intervention period, leaving a higher percentage of children with an often unclear diagnosis. This suggests that enhanced control of malaria may lead to a decrease in the positive predictive value of clinical symptoms for the diagnosis of malaria and a need for clinical caregivers to reassess empiric management of febrile illnesses in formerly malaria highly endemic zones when the prevalence of malaria declines. Other studies also suggest rapid declines in malaria after implementation of prevention and early treatment strategies, and indeed data specifically referring to declines in malaria admissions are beginning to be reported [16]. However, not all studies have shown that home based management is effective, highlighting the need for site specific outcome studies [11]. This study is unique in examining rural hospitalization patterns as well as laboratory markers for clinical disease and for reporting other types of admissions as malaria cases decline.

An important issue raised in this study is how to manage febrile illnesses when the prevalence of malaria declines and febrile illnesses are less likely to be due to malaria. This study found that with high malaria prevalence in the period prior to the intervention, clinicians were much more likely to be correct in attributing febrile illnesses to malaria than they were in the period following the intervention (80% vs. 48%), when laboratory-confirmed cases of malaria were less common. In much of sub-Saharan Africa's malaria-endemic regions, febrile illnesses are assumed to be malaria and are often treated only with antimalarial medications [17,18]. This can have devastating results for patients with other aetiologies of fever, in particular severe bacterial infections [19,20]. Indeed, in one large study in Tanzania diagnosis of malaria with negative laboratory examinations correlated with increased mortality unless antibacterial therapy was administered as well [21]. This suggests a significant problem with undiagnosed causes of fever and will require better diagnostic capabilities and data as well as fundamental alteration of approaches to treatment of febrile illnesses. Attempts to develop clinical algorithms for diagnosing malaria have had mixed results, and accuracy is dependent on local prevalence, which will in turn be altered by successful malaria control efforts [22,23]. The increased role of diagnostics, either slide microscopy or rapid diagnostic tests (RDTs) will need to be further explored as the epidemiology of febrile illnesses changes [24,25]. Over-treatment of malaria, as demonstrated in this study by the large number of children with negative slides who were given antimalarials, can become more problematic as prevalence drops. Over-treatment can lead to parasite drug resistance, inappropriate use of antimalarial medications, and inaccurate diagnosis and management of other febrile illnesses. Additionally, as malaria incidence declines children may become more severely ill if immunity is decreased from less frequent parasite exposure [26,27]. With further successes in malaria control, the issue of accurate diagnosis and treatment will become increasingly more critical in providing optimal care for patients with fever in resource-limited settings.

There are several possible limitations to this study, foremost of which is missing data as a result of the retrospective nature of the data collection. This study was based on existing records and reporting standards, and not specialized study protocols. It is unlikely that missing malaria thick smear data account for the observed decrease in laboratory-confirmed malaria during the intervention period given the relatively equal distribution of missing smear data between years. Furthermore, there were no identified systemic changes in data gathering or reporting between the intervention periods that would account for this association.

Haemoglobin levels, an indicator of disease severity, were higher in the post-intervention period. The trend towards higher haemoglobin levels for all children, and not solely those with laboratory-confirmed disease, is relevant in that lower haemoglobin is often a marker of repeat malaria infections in the community [28]. It is possible however, that the results of the haemoglobin analysis are biased by the significantly increased proportion of children receiving haemoglobin tests in post-intervention period. This increase in haemoglobin testing was likely due to ongoing intensive staff training on malaria protocols, including strict monitoring haemoglobin. If children who received haemoglobin tests tended to be sicker than children who were not tested in the pre-intervention period, this could potentially explain the observed increase in haemoglobin over time.

Confounding by other variables, such as changing hospital utilization patterns or rainfall, are unlikely to account for the observed decrease in laboratory-confirmed malaria. These reductions occurred during a period where admissions and service uptake for all other causes increased, and the number of paediatric non-confirmed malaria-like illness held constant. Similarly, the existence of a coincident decrease in infectious mosquitoes due to differences in weather conditions and rainfall is not substantiated by differences in rainfall or temperature between study seasons [29]. Children were significantly older in the post-intervention period, but this is likely due to the decreased incidence of malaria admissions, which are overwhelmingly predominant in younger children. Gender, however, was equally distributed between the study periods.

Many areas of future study are raised by this study, falling primarily in the two main categories of malaria control and management of febrile illnesses in resource-poor settings, especially as malaria prevalence falls. One of the primary needs in the first category is to undertake further analyses to assist with the continued refinement of malaria control programmes. The CHW model has been very successful and enhancement of their capabilities, such as the employment of rapid diagnostics for malaria and monitoring of disease at the local level, will be important. Additionally, it is not possible from this study to determine the relative contributions of mass LLIN distribution, early community-based treatment and in the future the effect of ACT. Better defining which interventions are most effective is also important for programme design, and studies are currently underway addressing optimal methods of prevention. Evaluation of the effect of extension of LLIN coverage to populations not considered at high risk for severe malaria may also further impact malaria prevalence. Indeed, there are data to suggest that increasing LLIN coverage to the entire community leads to even greater reductions in malaria in traditional target populations such as pregnant women and young children [30]. Continued and more sophisticated measures of outcomes on disease both at the local and hospital level will be important in measuring effective interventions and responding to changes in health needs over seasons and years [31]. Cost-effectiveness analyses may additionally be undertaken to assess the savings in both direct costs from hospitalization and lost productivity and indirect costs such as decreased school achievement and impaired cognitive development.

## Conclusion

The data for this study suggest that following intensive community-based prevention and early treatment programmes, there was a significant decline in admissions for malaria and improvement of laboratory markers of malaria disease at a time when admissions for all other causes increased. There was also an increase in the proportion of febrile illnesses diagnosed and treated as malaria despite negative laboratory studies, thus suggesting a need for clinicians to reassess management of febrile illnesses as malaria prevalence falls. This model additionally demonstrates the importance of government-sponsored programmes enjoying support by non-governmental organizations (NGOs) in effecting large-scale change in resource-limited settings. Continued monitoring over time and measurements of vector capacity and other variables that may impact disease prevalence, as noted above, will be necessary. However these data suggest that intensive community-based prevention and early treatment programmes can rapidly result in a reduction of severe paediatric malaria in rural Africa, and that government-NGO collaborations are an effective mechanism for implementing such programmes.

## Competing interests

The authors declare that they have no competing interests.

## Authors' contributions

AS worked on the malaria programme at Rwinkwavu, extracted and analysed data, and drafted the manuscript. JL was co-director of the Rwinkwavu malaria programme. PM was director of the home-based management programme at PIH. MF reviewed and edited the manuscript, provided the statistical analysis, and assisted in study design. BB assisted in data extraction and performed the mortality analysis. SS directed the paediatric programme and provided support for this study. MR directed Partners in Health Rwanda and provided support for the malaria programme and this study. CK is the head of PNILP, the national malaria control programme for Rwanda, and lead the LLIN and home-based management efforts. JD reviewed the manuscript extensively, provided guidance in study design, implementation, and statistical analysis, and helped draft the manuscript. All authors reviewed and approved the final version of the manuscript. All authors report no conflict of interest in this study.
